# Atypical temporal-scale-specific fractal changes in Alzheimer’s disease EEG and their relevance to cognitive decline

**DOI:** 10.1007/s11571-018-9509-x

**Published:** 2018-10-08

**Authors:** Sou Nobukawa, Teruya Yamanishi, Haruhiko Nishimura, Yuji Wada, Mitsuru Kikuchi, Tetsuya Takahashi

**Affiliations:** 1 Department of Computer Science, Chiba Institute of Technology, 2–17–1 Tsudanuma, Narashino, Chiba 275–0016 Japan; 2 Department of Management Information Science, Fukui University of Technology, 3–6–1 Gakuen, Fukui, Fukui 910–8505 Japan; 3 Graduate School of Applied Informatics, University of Hyogo, 7–1–28 Chuo-ku, Kobe, Hyogo 650–8588 Japan; 4 Department of Neuropsychiatry, University of Fukui, 23–3 Matsuokashimoaizuki, Eiheiji, Yoshida, Fukui, 910–1193 Japan; 5 Research Center for Child Mental Development, Kanazawa University, 13–1 Takaramachi, Kanazawa, Ishikawa 920–8640 Japan

**Keywords:** Alzheimer’s disease, EEG, Fractal analysis, Temporally specific fractality, Higuchi’s fractal dimension

## Abstract

Recent advances in nonlinear analytic methods for electroencephalography have clarified the reduced complexity of spatiotemporal dynamics in brain activity observed in Alzheimer’s disease (AD). However, there are far fewer studies exploring temporal scale dependent fractal properties in AD, despite the importance of studying the dynamics of brain activity within physiologically relevant frequency ranges. Higuchi’s fractal dimension is a widely used index for evaluating fractality in brain activity, but temporal-scale-specific characteristics are lost due to its requirement of averaging over the entire range of temporal scales. In this study, we adapted Higuchi’s fractal algorithm into a method for investigating temporal-scale-specific fractal properties. We then compared the values of the temporal-scale-specific fractal dimension between healthy control (HC) and AD patient groups. Our data indicate that relative to the HC group, the AD group demonstrated reduced fractality at both slow and fast temporal scales. Moreover, we confirmed that the fractality at fast temporal scales correlates with cognitive decline. These properties might serve as a basis for a useful approach to characterizing temporal neural dynamics in AD or other neurodegenerative disorders.

## Introduction

Recent advances in nonlinear analytic methods, applied to various neuroimaging modalities, have clarified the spatiotemporal dynamics of complex brain activity. Cortical synaptic weights have a log-normal distribution that can lead to spontaneous activity (Teramae et al. 2012). This activity constitutes the brain’s noisy internal state, with irregular neuronal spiking and a low average firing rate ($\approx 1$ Hz) even in the absence of sensory stimulation (Buzsáki and Mizuseki 2014). Feedback loops connecting neural populations at multiple hierarchical levels of cortical processing can produce corresponding recurrent patterns of brain activity (Fell et al. 1999). Therefore, brain activity is best modeled as a nonlinear dynamic process, which includes multiple coupling strengths following various distributions with heavy tails and feedback loops within and across multiple neural populations (Stam 2005; Teramae et al. 2012; Yamanishi et al. 2012; Strack et al. 2014; Fletcher and Wennekers 2016; Geminiani et al. 2017). Moreover, it has been demonstrated that the fluctuations and variability generated by this nonlinear dynamic process play an important role in the neural bases of cognitive function, aging, and psychiatric disorders. For example, Garrett et al. demonstrated that blood oxygen level-dependent (BOLD) signal variability was negatively correlated with age and positively correlated with cognitive function (Garrett et al. 2010, 2011). Mcintosh *et al.* also showed that a larger variability of response times in single-trial evoked electrical activity, as measured by electroencephalography (EEG), was associated with increased accuracy of recognition (McIntosh et al. 2008). Zhang et al. suggested that the spatiotemporal variability of BOLD signals reflects brain functions themselves, and is disturbed in psychiatric disorders such as schizophrenia, autism spectrum disorder, and attention-deficit hyperactivity disorder (ADHD) (Zhang et al. 2016).

Alzheimer’s disease (AD) involves three main types of changes: progressive central neuron death, neurofibrillary tangles, and senile plaques in widespread brain regions. The pathological progression leads to cortical disconnection, which reportedly alters the complex nonlinear behavior of the brain (Stam 2005; Delbeuck et al. 2003; Adeli et al. 2005b; Yang and Tsai 2013; Takahashi 2013; Bhat et al. 2015; Mammone et al. 2017). A variety of methods based on nonlinear dynamics have been applied to EEG data to characterize this alteration, including entropy analysis, correlation dimension, and omega-complexity (Yang and Tsai 2013; Takahashi 2013). Adeli et al. developed novel mixture markers and computational methods that, when combined with neural computing, chaos theory, and wavelet analysis, greatly increase the accuracy of diagnosis and detection of AD based on EEG signals (Adeli et al. 2005b, a).

A chaos/fractal-based approach (Kantz and Schreiber 2004) may be well-suited to analyzing nonlinearity in the brain. This is especially true when the alteration in AD is interpreted as a change in the deterministic properties of the nonlinear system. A growing number of studies utilizing this approach have demonstrated reduced fractality/chaoticity in AD. Using Haussdorff’s fractal dimension, Woyshville and Calabrese showed reduced complexity in occipital loci of subjects with AD in a resting condition (Woyshville and Calabrese 1994). Besthorn et al. reported a decreasing correlation dimension in EEG signals of patients with AD in an eyes-closed resting state (Besthorn et al. 1995). Jelles et al. also observed reductions of this correlation dimension in three conditions: eyes closed, eyes open, and during an arithmetic task (Jelles et al. 1999). Other methods, including the use of fractal dimensions and the maximum Lyapunov exponent, have replicated these reductions (Jeong 2004; Abásolo et al. 2008; Zappasodi et al. 2014; Smits et al. 2016; Al-nuaimi et al. 2017).

EEG dynamics reflect different functions for each physiologically relevant temporal scale. For example, perception is associated with gamma band dynamics, cognition with the beta band, and memory with the theta band (Klimesch et al. 2007). Also, because of complex activity generated by nonlinear dynamics, the power spectrum does not always conform to a simple power law distribution over the entire frequency range (Ferree and Hwa 2003; Miller et al. 2009). Thus, it is important to measure complexity for specific temporal scales.

In fact, the temporal-scale dependence of complexity in EEG signals has previously been studied in AD. In our earlier review (Takahashi 2013), we introduced work based on multiscale entropy, in which it was found that EEG signals in AD demonstrated lower complexity at smaller temporal scales and higher complexity at larger temporal scales (Escudero et al. 2006; Park et al. 2007; Mizuno et al. 2010). Adeli et al. demonstrated that alterations in EEG signals in AD are localized to certain frequency bands (delta and theta bands in an eyes-open condition; delta, theta, and alpha bands in an eyes-closed condition) by using the maximum Lyapunov exponent and correlation dimension in band-specific EEG signals analyzed by wavelet transformation (Adeli et al. 2008).

Higuchi’s fractal dimension, which is defined by the power law for the length of a time series as a function of the temporal scale (Higuchi 1988), has been widely used to evaluate fractality in brain activity (Smits et al. 2016; Accardo et al. 1997; Jeong et al. 2001; Gómez et al. 2009; Nishimura et al. 2008; Zappasodi et al. 2014). Higuchi reported that different power laws, i.e., different fractal dimensions, often appear for different temporal scales when his fractal algorithm is applied to an experimental time series (Higuchi 1988). However, these temporal scale specific characteristics are ignored in the final result, because the algorithm averages over the entire temporal scale range. For example, Smits et al. showed that Higuchi’s fractal dimension converges with increasing maximum value of temporal-scale (Smits et al. 2016). To detect the temporal-scale specific characteristics, Higuchi proposed a method for calculating the dimension at a specific temporal scale (herein described as the temporal-scale-specific fractal dimension) (Higuchi 1988). However, the temporal-scale-specific fractal dimensions in band-specific ranges of temporal scale have not been investigated in EEG signals. An alternative approach by Adeli et al. introduced a novel method for calculating fractal dimensions, including Higuchi’s fractal dimension, by using wavelet transformation to divide EEG signals into frequency bands (Adeli et al. 2007). Their approach has produced insights into neuropsychiatric disorders such as autism spectrum disorder, seizure activity and epilepsy (Ahmadlou et al. 2010; Adeli et al. 2007). Ahmadlou *et al.* investigated band-specific fractal dimensionality, and found decreased fractality in the beta band of the AD EEG using this approach (Ahmadlou et al. 2011).

To identify the temporal scale characteristics of the complex time-series of the AD EEG, we previously attempted to calculate the temporal-scale-specific fractal dimension of AD (Nobukawa et al. 2017). However, the parameter settings needed to estimate temporal-scale-specific fractal dimension and the relationship between temporal-scale-specific fractal dimension and cognitive function have not been clarified. In this study, following up on the results of our previous work (Nobukawa et al. 2017), we first derived a parameter set that can be used to estimate the temporal-scale-specific fractal dimension. Second, we evaluated the temporal scale and regional characteristics of this dimension in healthy control (HC) subjects and AD patients. Third, we investigated the correlation between the temporal-scale-specific fractal dimension and cognitive function, as estimated by the Mini Mental State Examination (MMSE) score, (Folstein et al. 1975) in the AD group.

## Materials and methods

### Participants

In this study, we used the same participants who were examined in our earlier study (Mizuno et al. 2010). The patient group consisted of 16 subjects diagnosed with AD (mean age 57.5 years, age range 43–64 years, SD of age: 4.7 years, 11 female), and 18 age-matched and sex-matched healthy controls (HC) (mean age 59.3 years, age range 55–66 years, SD of age: 5.3 years, 11 female), as shown in Table 1. All subjects provided written informed consent before the research. Each AD subject was assessed with the Functional Assessment Stages Test (FAST) (Reisberg et al. 1986) and a Japanese version of the MMSE (Folstein et al. 1975). According to the FAST assessments, 3 patients had mild (FAST 3), 7 moderate (FAST 4), and 6 slightly severe dementia (FAST 5). Their MMSE scores were distributed in the ranges from 10 to 26 (mean score: 15.5, SD of score: 5.3).

**Table 1:** Physical characteristics of subjects (values represent mean (SD, range))

	HC subjects	AD subjects	*p* values
Male/female	7/11	5/11	0.72
Age (years, range)	59.3 (5.3, 55–66)	57.5 (4.7, 43–64)	0.31
MMSE score	NA	15.5 (4.7, 10–26)	

### EEG recordings

Recording and pre-processing of the EEG data were accomplished as reported in our previous study (Mizuno et al. 2010). Briefly, the subjects were studied while seated in an electrically shielded, soundproofed, and light-controlled recording room. Standard scalp electrodes were placed according to the International 10–20 System. EEG was recorded with an 18-channel electroencephalogram (EEG–4518, Nihon–Koden, Tokyo, Japan) at 16 electrode sites: Fp1, Fp2, F3, Fz, F4, F7, F8, C3, C4, P3, Pz, P4, T5, T6, O1 and O2, referenced to physically linked ear lobe electrodes. Eye movements were monitored by means of bipolar electro-oculography (EOG). EEG signals were recorded with 200 Hz sampling frequency with a time constant of 0.3 s, and a 1.5–60 Hz bandpass filter. Line noise was eliminated by a 60 Hz notch filter. The impedance of electrode/skin conductance was maintained at less than 5 k$\Omega$ for each electrode. EEG signals were recorded for 10–15 min for each subject with the eyes closed. The subjects were observed via a video monitoring system. The state of vigilance of the subject was visually inspected during recording using the EEG traces to ensure that only epochs of eyes-closed wakefulness (and not light sleep) were analyzed. When the subjects appeared to be drowsy, they were asked to open their eyes and verbally reminded to avoid drowsiness. Selection of segments recorded during eyes-closed wakefulness was performed by visual inspection of EEG and EOG recordings. A subject was considered to be fully awake when alpha activity appeared predominantly over the posterior regions, with concurrent fast eye movements in the EOG channel (Wada et al. 1996).

The data were stored on an optical disk for off-line analysis. Other pre-processing steps (e.g., filtering, artifact removal or data reconstruction) were avoided, because they might have disrupted the intrinsic dynamics of the data. Epochs without artifacts were selected by visual inspection. To provide adequate information for analysis of long-range temporal dynamics, we initially prepared a single continuous artifact-free 60-s (12,000 data points) epoch during the eyes-closed resting condition. From this dataset, 1000 data points at the beginning and end were removed to avoid interference by the 1.5–60 Hz bandpass filter. Finally, Higuchi’s fractal dimension was calculated on the continuous 50-s (10,000 data points) epoch.

### Fractal analysis

For the fractal analysis of EEG data, we used Higuchi’s fractal algorithm (Higuchi 1988). If the EEG signal at a site has fractal characteristics, similar patterns arise at different temporal scales. To quantify this similarity, first, using scale *k*, the EEG data *X*(*t*)($0,1,\ldots ,T$) is down-sampled to $\{X(m),X(m+k),X(m+2k),\ldots ,X(m+[(T-m)/k]k)\}$ where [ ] indicates the Gauss symbol and *m* is the first sample. The length of *X* at each scale *k* is defined by

1 $$\begin{aligned}&L(k) \equiv \frac{1}{k} \left[ \frac{T-1}{\left[ \frac{T-m}{k}\right] \ldots k}\right. \nonumber \\&\left. \qquad \cdot \left( \sum _{i=1}^{\left[ \frac{T-m}{k}\right] } | X(m+i\cdot k)- X(m+(i-1)\cdot k)|\right) \right] . \end{aligned}$$

Here, if the time series of *X*(*t*) has the fractal dimension *D*, $\langle L(k)\rangle$ ($\langle \cdot \rangle$: average over *m*) obeys

2 $$\begin{aligned} \langle L(k)\rangle \propto k^{-D}. \end{aligned}$$

In this study, we estimated the *D* value in the range of $$k_\text {min} \le k \le k_\text {max}$$. Concretely, *D* can be derived as the slope of $$\{(\log (L(k_\text {min})),\log (1/k_\text {min})),(\log (L(k_{\text {min}+1})),$$$$\log (1/k_{\text {min}+1})),\ldots, (\log (L(k_\text {max}))$$$$,\log (1/k_\text {max})) \}$$, using the linear least-squares method.

### Power analysis

Along with the analysis for temporal-scale-specific fractal dimension, we also performed power spectral analysis as in conventional EEG analyses. The power spectrum density (PSD) (dB/Hz) was estimated using a fast Fourier transform. A Hanning window was applied to the 60 s time-series for the calculation.

### Statistical analysis

Group differences were analyzed using independent two-tailed *t* tests. Because the temporal-scale-specific fractal dimension values were found to have a skewed distribution, we log-transformed them to obtain an approximately normal distribution. Associations between temporal-scale-specific fractal dimensions and cognitive function estimated by MMSE score were evaluated using Pearson’s correlation coefficient. The Benjamini–Hochberg false discovery rate (FDR) correction was applied for multiple comparisons. For sensor-wise group comparisons, $q<0.05$ was used for fractal dimensions calculated over the entire range of temporal scales ($D_\text {entire}$; 16 *p* values), for temporal-scale-specific fractal dimensions ($D_\text {slow}$, $D_\text {alpha}$, and $D_\text {fast}$; 48 *p* values), and for PSD (896 *p* values: 56 frequency points ($5--60$ Hz, width of bin is 1.0 Hz) $\times$ 16 electrodes).

Additionally, repeated measures analysis of variance (ANOVA), with group (HC vs. AD) as between-subjects factor and node (16 nodes of Fp1-O2) as within-subjects factor, was performed to test for group differences at each frequency band. The Greenhouse-Geisser adjustment was applied to the degrees of freedom, and a two-tailed $\alpha$ level of 0.05 was considered statistically significant.

## Results

### Power analysis

**Fig. 1:**
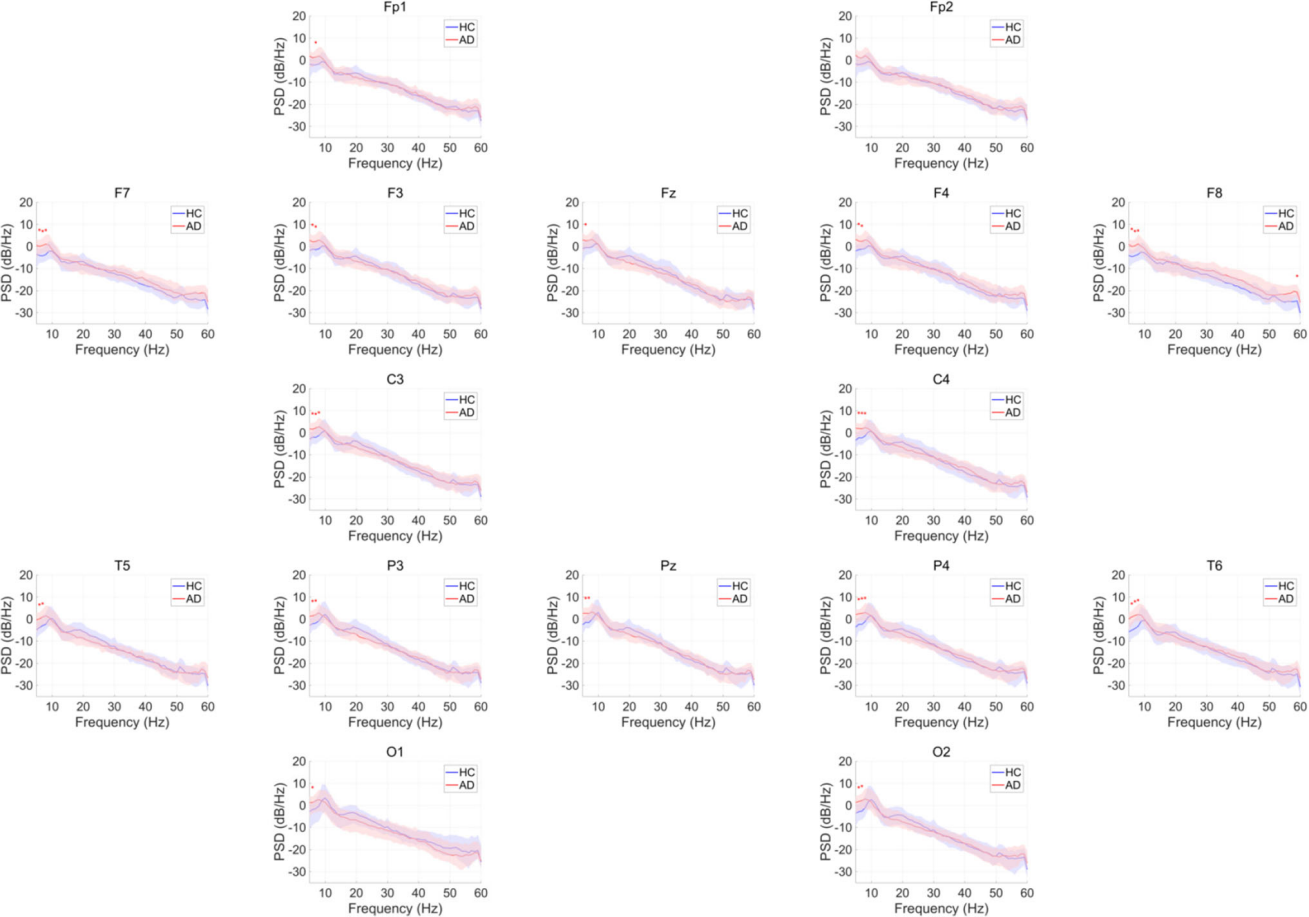
Power spectrum of EEG data for HC subjects and AD subjects (*HC* healthy controls, upper, *AD* Alzheimer’s disease). Solid lines and shaded area represent mean and standard deviation in each group. The red $*$ indicate differences that are significant after adjustment for false discovery rate (FDR) $q<0.05$, respectively. (Color figure online)

To observe the differences in power spectrum between EEG signals of HC and AD, as shown in Fig. 1, the average PSD of the HC group and AD group were calculated for each electrode. Significant enhancement of PSD in the delta–theta bands (2–8 Hz) ($q < 0.05$) were identified in the AD group across all electrodes, which is consistent with previous findings of slowing wave of AD (Ishii et al. 2017).

### Temporal-scale-specific fractal dimension

#### Parameter setting for temporal-scale-specific fractal dimension

**Fig. 2:**
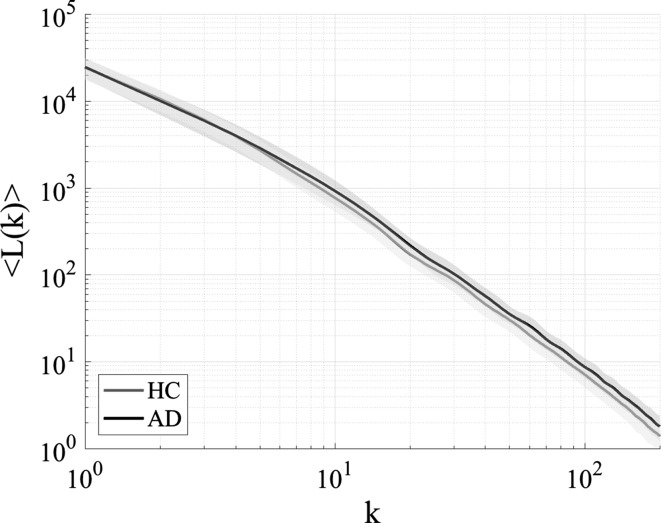
Dependence of $\langle L(k)\rangle$ on temporal scale *k* at Fz in the HC and AD groups

**Fig. 3:**
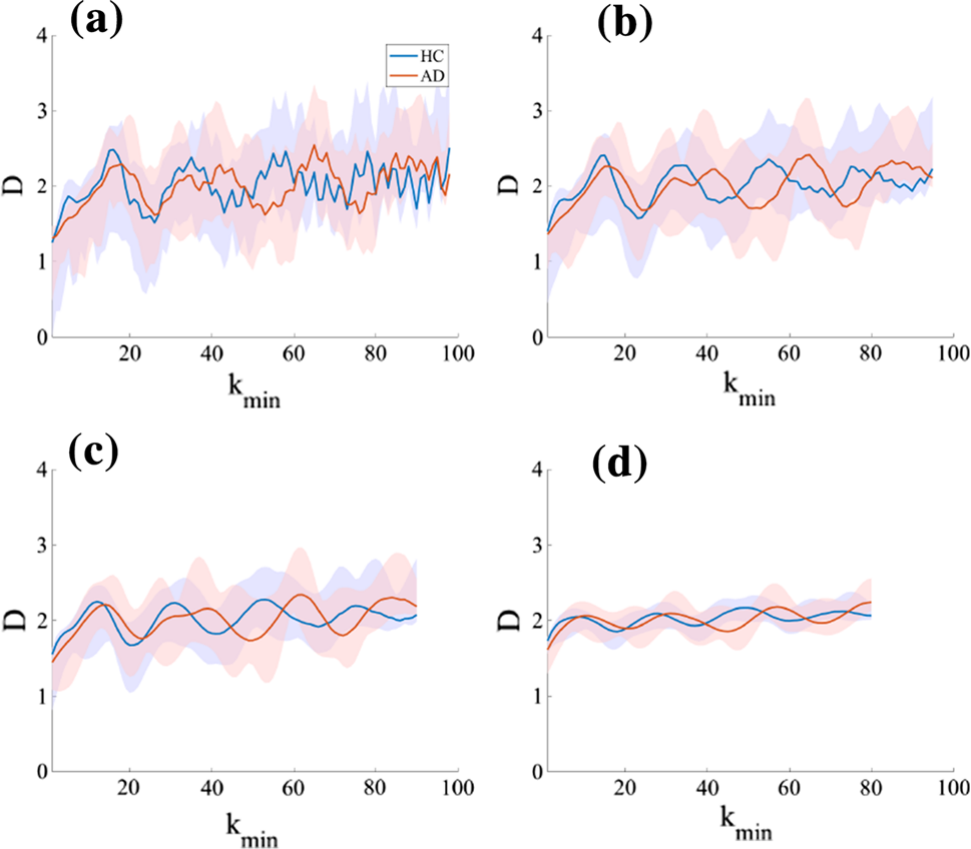
Dependence of the temporal-scale-specific fractal dimension *D* on $k_\text {min}$ at Fz in the HC and AD groups. **a**$k_\text {max}-k_\text {min}=2$. **b**$k_\text {max}-k_\text {min}=5$. **c**$k_\text {max}-k_\text {min}=10$. **d**$k_\text {max}-k_\text {min}=20$

**Fig. 4:**
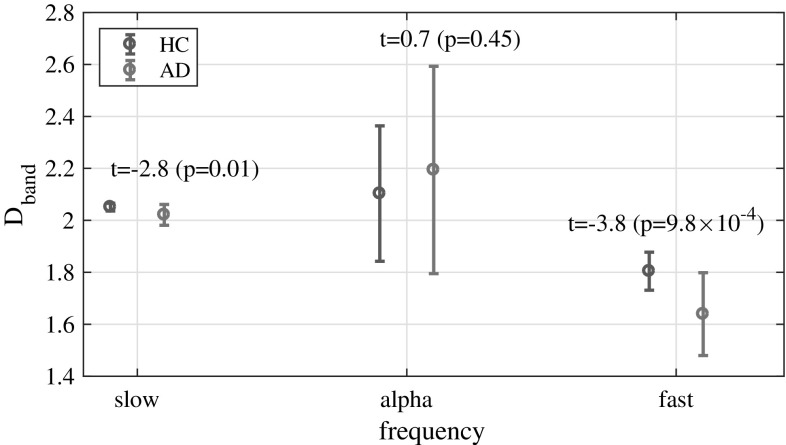
Temporal-scale-specific fractal dimension $D_\text {slow,alpha,fast}$ in the *k* ranges given by Table 2 at Fz in the HC and AD groups

We evaluated the range of *k* to derive temporal-scale-specific fractal dimension. Figure 2 shows the typical example of dependence of $\langle L(k)\rangle$ on *k* for $1\le k\le 200$ in the case of Fz node from subjects in the HC and AD groups. Figure 3 shows the slopes of $\langle L(k)\rangle$ between $k_\text {min}$ and $k_\text {max}$ given by the $\langle L(k)\rangle$ values appearing in Fig. 2 as a function of $k_\text {min}$ in the cases of $k_\text {max}-k_\text {min}=2,5,10,20$ cases. For $k_\text {max}-k_\text {min}\ge 10$, a smoothed dependency of *D* on $k_\text {min}$ is obtained. Therefore, using the ranges $k_\text {max}-k_\text {min} \ge 10$, we calculated temporal-scale-specific fractal dimensions for the slow, alpha, and fast bands in the HC and AD groups (see Table 2). Here, the $k_\text {min,max}$ are derived from $[1/f_\text {max,min}]$ ($f_\text {max,min}$ Hz are upper/lower limits of frequency band). In the case of division into 5 bands (delta, theta, alpha, beta, and gamma band), $k_\text {min}$–$k_\text {max}$ becomes less than 10 scales. Therefore, we used 3 bands instead (slow, alpha, and fast). Figure 4 shows the resulting temporal-scale-specific fractal dimensions $D_\text {slow,alpha,fast}$ at Fz in the HC and AD groups. From this result, the temporal-scale-specific nature can be confirmed in both groups.

**Table 2:** Ranges of *k* and fractal dimension corresponding to each frequency range

Frequency range	$k_\text {min}-k_\text {max}$	Temporal-scale-specific fractal dimension
Entire range (1.5–60 Hz)	3–133	$D_\text {entire}$
Slow range (2–8 Hz)	25–100	$D_\text {slow}$
Alpha range (8–13 Hz)	15–25	$D_\text {alpha}$
Fast range (13–60 Hz)	3–25	$D_\text {fast}$

#### Comparison between HC and AD by temporal-scale-specific fractal dimension

**Fig. 5:**
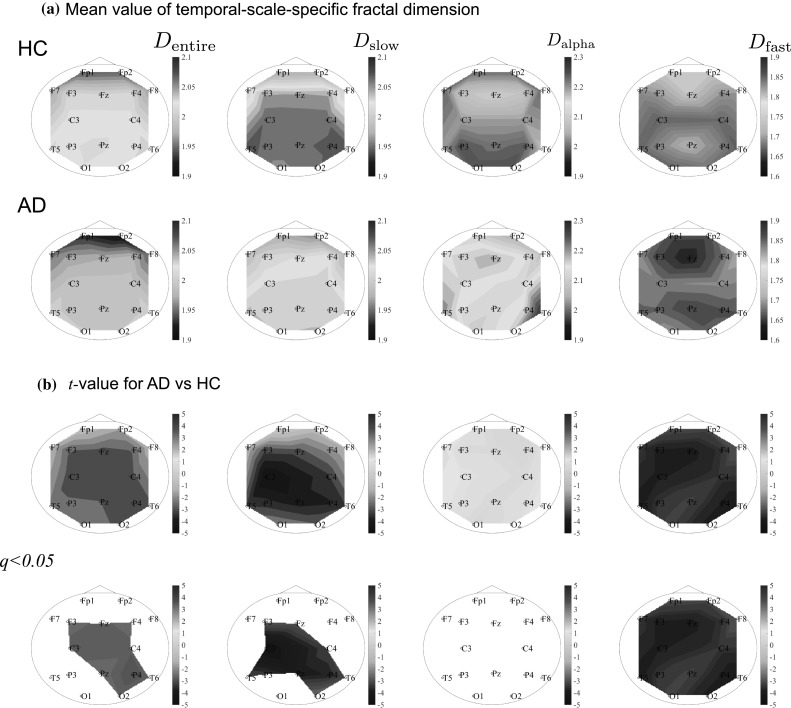
**a** Mean value of temporal-scale-specific fractal dimension in Alzheimer’s disease patients (AD) and healthy controls (HC). **b***t* values for group comparison of fractal dimension between AD and HC. Cold colors indicate that fractal dimensions are lower in the AD group than in the HC group, while warm colors indicate the opposite. Upper panels indicate *t* values at all nodes. Lower panels are cases meeting the FDR criterion $q<0.05$

We calculated the fractal dimension within the entire temporal scale range and for specific temporal ranges in the HC and AD groups, as shown in Fig. 5a, b. Table 3 summarizes the results of the ANOVAs on $D_\text {entire}$ and $D_\text {slow},D_\text {alpha},D_\text {fast}$. Testing for group differences between HC and AD revealed an effect of group for the entire, slow and fast ranges. In post-hoc *t* tests, the $D_\text {entire}$ values for the AD group were lower than the values for the HC group. Although group $\times$ node interactions were not confirmed in ANOVAs, the *t* values for nine nodes (F3, F4, Fz, C3, C4, P4, Pz, T6, O2) met the FDR criterion ($q<0.05$), as shown in Fig. 5b, bottom panels. The temporal-scale-specific fractal dimensions for the AD group were low in the slow and fast bands. In particular, although group $\times$ node interactions were not confirmed in ANOVAs, the *t* values at F3, Fz, C3, C4, T5, T6, P3, P4, Pz and O2 in the slow range, subjected to the FDR criteria ($q<0.05$), were significantly lower. Those at all nodes were also significantly lower in the fast range ($q<0.05$).

**Table 3:** Repeated measures ANOVA results for temporal-scale-specific fractal dimensions comparing the AD and HC groups for each temporal ranges

Frequency band	Group effect	Group $\times$ node
Entire band	$\mathbf {F}=\mathbf {5.3,p}=\mathbf {0.02}$	$F=0.56,p=0.59$
Slow band	$\mathbf {F}=\mathbf {8.0,p}=\mathbf {8.0}\times \mathbf {10}^{\mathbf {-3}}$	$F=0.97,p=0.39$
Alpha band	$F=0.8,p=0.35$	$F=0.7,p=0.047$
Fast band	$\mathbf {F}=\mathbf {14.7,p}=\mathbf {5.5}\times \mathbf {10}^{-\mathbf {4}}$	$F=1.0,p=0.38$

For clarity, comparisons with $p<0.05$ are shown in bold

#### Correlations of temporal-scale-specific fractal dimension with MMSE score in AD

**Fig. 6:**
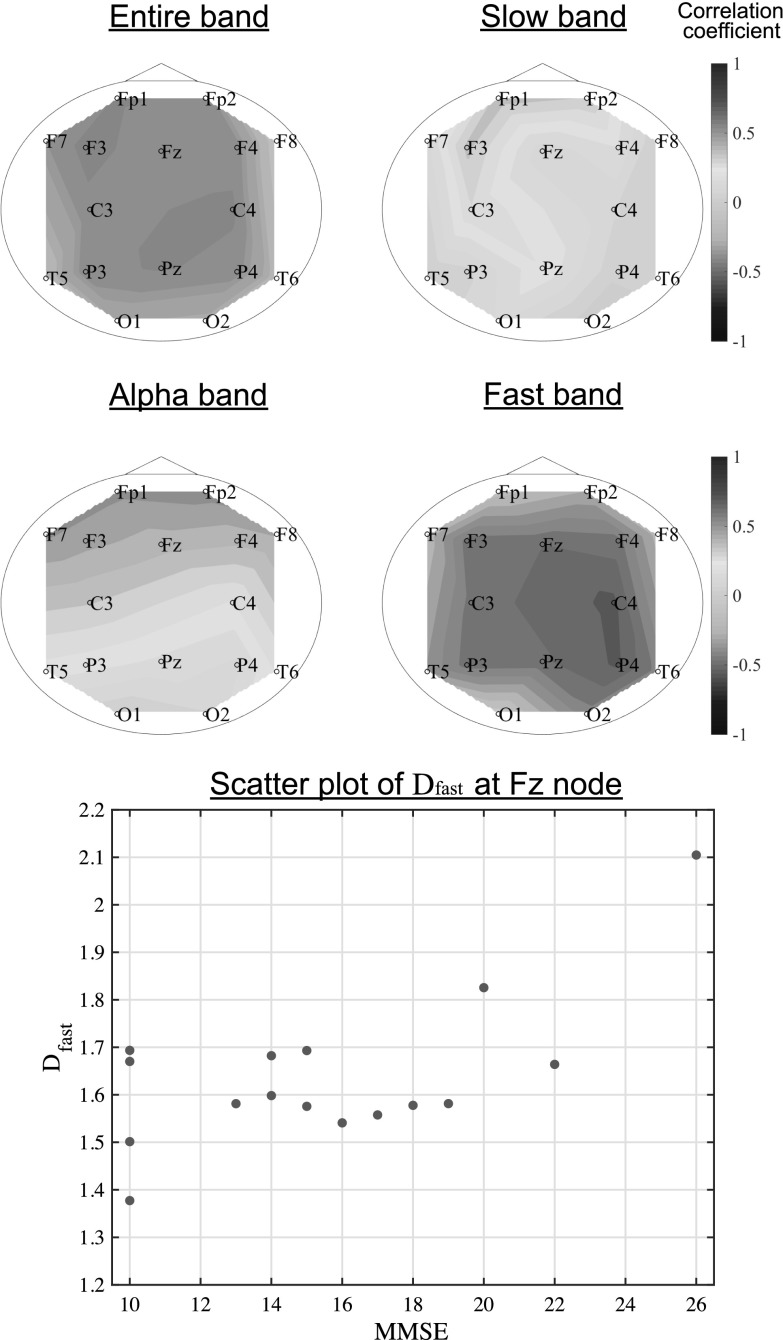
Correlation between temporal-scale-specific fractal dimension and Mini Mental State Examination (MMSE) score in Alzheimer’s disease (AD) subjects (upper panels). Scatterplot for the AD subjects of $D_\text {fast}$ at Fz node and MMSE score (lower panel)

The upper parts of Fig. 6 show the correlations between the fractal dimension and MMSE scores in the AD group across the range of recording sites. The $D_\text {entire}$ correlation values were all greater than or equal to 0.4. For the specific band case, $D_\text {fast}$ showed higher correlations ($\gtrsim 0.6$) than the entire range and the other temporal-scale-specific fractal dimensions. The lower part of Fig. 6 shows a scatterplot of $D_\text {fast}$ at Fz node and MMSE score for the AD subjects. High correlations were also observed from this result.

#### Validity of the epoch length of EEG signals

**Fig. 7:**
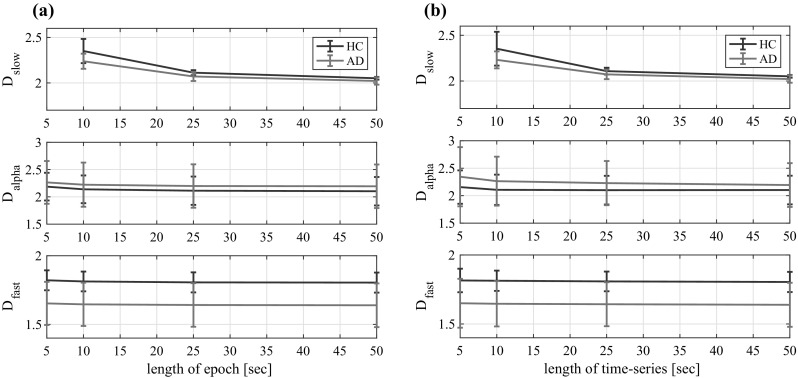
**a** Variation of fractality against changing epoch length at Fz in HC and AD groups. For $D_\text {slow}$ in the 5 s case, the epoch length was too short to allow calculation of the temporal-scale-specific fractal dimension. **b** Variation of fractality against shortening evaluation time-series length at Fz in HC and AD group

To check the validity of the epoch length of EEG signals for temporal-scale-specific fractal dimension, Fig. 7a shows the variation of fractality against the changing epoch length at Fz in the HC and AD groups. Here, the temporal-scale-specific fractal dimensions in segmented epochs were averaged over the full 50 s. All temporal-scale-specific fractal dimensions converged in the range $\gtrsim 30$ s. Therefore, the epoch length of 50 s that was used in the analyses above is valid for all temporal-scale-specific fractal dimensions.

We also evaluated the fractality using shorter evaluation time-series. In Fig. 7b, the variation of fractality resulting from shortening the evaluation time-series length is represented. The first epoch in a 50 s time-series was used for each length case. It is noteworthy that the values $D_\text {fast}$ maintained significant differences between AD and HC, even when the length of the time-series was short, i.e., $t=-3.4$ ($p=2.5 \times 10^{-3}$) at 5 s.

## Discussion and conclusion

In this study, we introduced a new approach that captures the temporal-scale-specific EEG fractal properties of AD patients. The results indicate that when fractality was integrated across the full range of temporal scales, the AD group exhibited reduced fractality. When temporal-scale-specific fractal properties were analyzed, reduced fractality were again observed. Specifically, in the AD group, reduced fractality was observed for faster frequency ranges (the beta and gamma range). Moreover, by evaluating the relationship between cognitive function measured by MMSE and the temporal-scale-specific fractal dimensions, we confirmed that the fractality at faster temporal scales correlates with cognitive decline. This reduced fractality can be detected even when the length of the time-series is small.

Reduced fractality is associated with reduced complexity, which is consistent with the well-established hypothesis (Stam 2005; Jeong 2004) that EEG signals in AD exhibit less complexity. EEG oscillations on the slow temporal scale are mainly determined by global regional coupling (Tononi et al. 1994; Friston et al. 1995). We have previously reported the value of multiscale entropy, which captures EEG complexity on multiple temporal scales (Mizuno et al. 2010). In this study, we found reduced EEG complexity in a scale-dependent manner that agrees well with our recent findings.

Stam et al. used a graph-theoretical analysis to argue that the decreasing functional connectivity in beta bands is related to cognitive function (Stam et al. 2009). Our result shown in Fig. 6, that is, high correlation between $D_\text {fast}$ and the MMSE score, is consistent with their finding. Also, studies of neurotransmitter changes in AD reported that dysfunction of the gamma- aminobutyric acid (GABA) signaling system leads to reduced oscillatory gamma band activity (Nava-Mesa et al. 2014; Govindpani et al. 2017; Calvo-Flores Guzmán et al. 2018). Thus, the temporal-scale-specific fractal characteristics of AD in the fast bands might reflect these changes in fast band activity. Ahmadlou et al. also investigated band-specific fractal dimensionality (Ahmadlou et al. 2011). They divided EEG signals into frequency bands by wavelet analysis before calculating Higuchi’s fractal dimension. Interestingly, despite methodological difference, they reported decreased beta band fractality across widespread brain regions, which is similar to our finding. However, this subdivision approach restricts frequency components, which may lead to perturbation of fractal properties determined by Eq. (2). If this type of band-pass filter is applied to EEG signals in our proposed method, due to the disturbance of the $\langle L(k)\rangle$ distribution, the significant difference in fractality is abolished (see appendix). In our proposed method, this disturbance can be avoided by using EEG signals composed of the entire set of frequency components.

More interestingly, the fast temporal-scale-specific fractal dimension converges and shows significant differences between HC and AD even with a short evaluation time-length of 5 s. However, the measure for the slow scale range does not show a significant difference, because it requires a longer time-series for evaluation. Therefore, the fast temporal-scale-specific fractal dimension might be a useful biomarker of AD that can be utilized even with a short time-series of EEG.

Several limitations of this study must be considered. Higuchi’s fractal dimension characterizes variation as a function of the *k* range. To characterize temporal-scale-specific properties, in this study we utilized *k* ranges corresponding to the conventional functional frequency bands: slow (delta/theta), alpha band, and fast band (beta/gamma). However, these ranges might need to be optimized for specific brain functions and diseases. This type of optimization should be considered in future work. Regarding cognitive functions, we evaluated the relationship between the temporal-scale-specific dimension and MMSE score in AD. However, due to the ceiling effect for the MMSE, a wide range of cognitive functions cannot be dealt with. Therefore, in future work, we should consider the relationship with other cognitive tests, while expanding the pool of healthy subjects. It is also important to evaluate the temporal-scale-specific fractal dimension in subjects performing cognitive tasks (rather than resting).

In conclusion, the AD group showed temporal-scale-specific reduced fractality and this reduced fractality was associated with cognitive decline. These findings highlight the potential utility of examining temporal-scale-specic fractality of EEG signals in diagnosing AD and evaluating disease severity. Additionally, the possible diagnostic utility of our method was confirmed even with short data sets, which is advantageous in a clinical setting. Although several limitations need to be clarified, characterizing temporal-scale specific fractal properties in neurophysiological data may serve as a powerful complementary approach for diagnosing neurodegenerative diseases.

## Figures and Tables

**Fig. 8:**
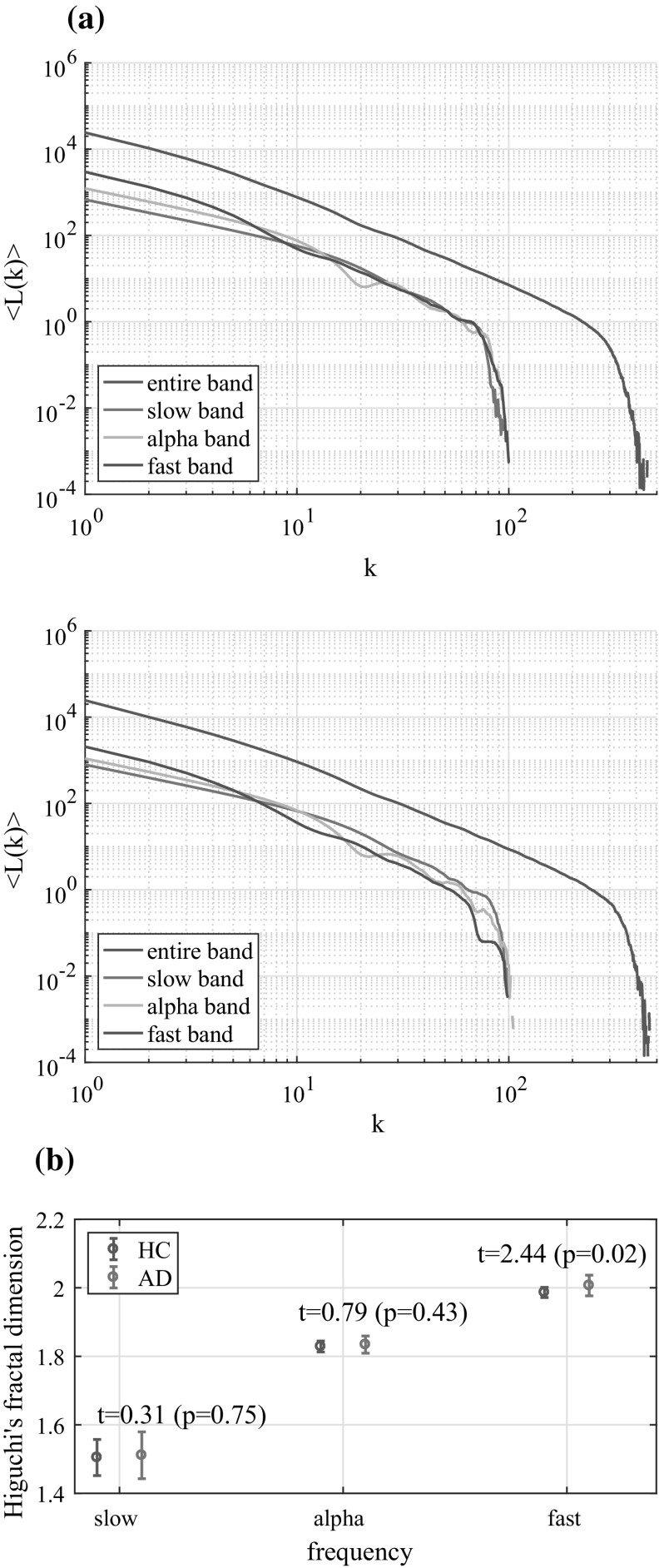
**a** Dependence of the mean value $\langle L(k)\rangle$ of HC (upper) and AD (lower) groups on *k* at Fz in the entire (1.5–60 Hz) (corresponding to Fig. 2), slow (2–8 Hz), alpha (8 – 13 Hz), and fast (13–60) band cases. **b** Higuchi’s fractal dimension for each distribution of $\langle L(k)\rangle$ in $1\le k\le 35$

## Appendix

To estimate the temporal-scale-specific fractal dimensions, we used the $\langle L(k)\rangle$ distribution for EEG signals composed of the entire frequency range (1.5–60 Hz) in this study. Here, we investigated the influence on the $\langle L(k)\rangle$ distribution of adopting band-pass filters for the slow, alpha, and fast bands. Figure 8a shows the dependence of the mean value $\langle L(k)\rangle$ of the HC and AD group on *k* at Fz in the entire (1.5–60 Hz), slow (2–8 Hz), alpha (8–13 Hz), and fast (13–60) band cases. The value for $\langle L(k)\rangle$ in entire band follows a power law in $k\,\lesssim\,200$. However, the value of $\langle L(k)\rangle$ for both groups in the slow, alpha, and fast bands does not obey the power law in the range $k \gtrsim 50$.

In our proposed method, the temporal-scale-specific fractal properties were derived using scale-specific *k* ranges. Instead of the scale-specific ranges, by the above distribution of $\langle L(k)\rangle$ for band-specific time-series, the temporal-scale-specific fractal properties were estimated as shown in Fig. 8b. Here, the Higuchi fractal dimension was estimated with the common $k_\text {min,max}=1,35$. $k_\text {max}$ was used as the minimum right edge of the distribution of $\langle L(k)\rangle$ in all subjects. As a result, the significant group differences observed with our method (see Fig. 5) disappeared. Therefore, the temporal-scale-specific fractal properties cannot be extracted by the band-pass filtering process, due to the disruption of the power law of $\langle L(k)\rangle$.
